# Observations on the Case of W. O.

**Published:** 1815-01

**Authors:** R. N. Starr

**Affiliations:** Late Assistant-Surgeon 48th Regt. Barnsley, Yorkshire


*For the Medical and Physical Journal.*

*Observations on the Case of W. O.;* by R. N. STARR, Surgeon, of Barnsley, Yorkshire.

ON perusing your Journal for this month, I observed a communication signed "W. O." respecting a syphilitic or supposed syphilitic complaint he has laboured under for some time. It appears the gentleman had contracted chancres, which were succeeded by buboes; mercury had been freely used, without affecting the system; in the mean time, the chancres healed, and after a lapse of three years, the ulcers in the groin also healed. It appears after this he was affected with nocturnal pains in the arms, thighs, and legs, more particularly of the tibia, with nodes on the latter. From these symptoms, I am inclined to coincide with the opinion of the medical gentleman who attended him in his passage to England, that the disease is evidently secondary syphilis, and most likely to be relieved by mercury. I have seen several cases, the symptoms of which resembled those of this gentleman, and which all gave way to the use of mercury. I should, therefore, recommend him to consult some eminent surgeon, who will, no doubt, allow the mercury a fair trial, which, in my opinion, it has not yet had. R. N. STARR,

*Late Assistant-Surgeon 48th Regt.*

*Barnsley, Yorkshire; Dec.* 5, 1814.

